# Establishment of a system evaluating the contractile force of electrically stimulated myotubes from wrinkles formed on elastic substrate

**DOI:** 10.1038/s41598-022-17548-7

**Published:** 2022-08-15

**Authors:** Hiroki Hamaguchi, Tsubasa S. Matsui, Shinji Deguchi, Yasuro Furuichi, Nobuharu L. Fujii, Yasuko Manabe

**Affiliations:** 1 Department of Health Promotion Sciences, Graduate School of Human Health Sciences, Tokyo Metropolitan University, 1-1 Minami-Osawa, Hachioji, Tokyo 192-0397 Japan; 2 Division of Bioengineering, Graduate School of Engineering Science, Osaka University, 1-3 Machikaneyama, Toyonaka, Osaka 560-8531 Japan

**Keywords:** Biochemical assays, Experimental models of disease

## Abstract

Muscle weakness is detrimental not only to quality of life but also life expectancy. However, effective drugs have still not been developed to improve and prevent muscle weakness associated with aging or diseases. One reason for the delay in drug discovery is that no suitable in vitro screening system has been established to test whether drugs improve muscle strength. Here, we used a specific deformable silicone gel substrate to effectively and sensitively evaluate the contractile force generated by myotubes from wrinkles formed on the substrate. Using this system, it was found that the contractile force generated by an atrophic phenotype of myotubes induced by dexamethasone or cancer cell-conditioned medium treatment significantly decreased while that generated by hypertrophic myotubes induced by insulin-like growth factor-1 significantly increased. Notably, it was found that changes in the index related to contractile force can detect atrophic or hypertrophic phenotypes more sensitively than changes in myotube diameter or myosin heavy chain expression, both commonly used to evaluate myotube function. These results suggest that our proposed system will be an effective tool for assessing the contractile force-related state of myotubes, which are available for the development of drugs to prevent and/or treat muscle weakness.

## Introduction

Muscle atrophy, a condition of decreased muscle mass, is caused by aging^1^, physical inactivity^2^, and cancer cachexia^3^, which results in decreased muscle strength, followed by impaired physical function. Muscle mass has been generally considered a strong predictor of physical function, but this is not always the case for the elderly^4^. In contrast, muscle strength is strongly related to physical function, and impaired physical function reduces not only quality of life but also life expectancy^5,6^. Therefore, the development of drugs for the treatment of muscle atrophy that prevent and/or improve muscle weakness has long been desired.

In light of this objective, researchers in this field have recently been searching for drug targets involved in the induction of muscle atrophy. For example, myostatin, which inhibits muscle mass increase via activin receptor type II (ActRII), has been the focus of much research as an effective drug target^7,8^. Several compounds which inhibit ActRII were investigated, to see whether they might ameliorate muscle atrophy^9,10^. Some of these compounds were shown to restore muscle mass, with potential to become a treatment, but none have achieved the goal of improving muscle strength and physical function in clinical trials. These results led to a new perspective on further research, i.e., the importance of assessing muscle strength when screening potential drugs to treat muscle atrophy.

In general, the first step for evaluating drug candidates requires an in vitro screening assay that allows comprehensive testing of a large number of drugs, genes, or proteins. Myotubes, differentiated skeletal muscle culture cells, are frequently used to study skeletal muscle diseases. To assess whether a compound prevents muscle atrophy in cultured cells, conventional measurements include the myotube diameter as a muscle mass indicator and the expression levels of proteins involved in muscle contractile function such as myosin heavy chain (MHC). Although these methods are well established, they do not directly assess muscle strength. Indeed, it was reported that increasing the diameter of artificial skeletal muscle constructs with epigenetic drugs does not necessarily change muscle contractile force^11^. Moreover, the expression of certain proteins such as MHC is not an absolute indicator of muscle strength. Since contractile force (i.e., muscle strength) is a more direct indicator of muscle function, drug screening for assessing muscle atrophy requires assessment of myotube contractile force.

Several methods for the evaluation of cellular force have already been reported. For example, traction force microscopy (TFM) is a conventional method for measuring cellular traction force^12^. However, TFM typically requires the use of confocal microscopy and the acquisition of reference distributions of fluorescent microbeads, which is extremely laborious. Existing methodologies other than TFM also have some limitations in terms of throughput, which largely limits their use as high-throughput assays for evaluating changes in cellular force. For example, a micropillar array method requires hydrophilic treatment as well as extracellular matrix printing on top of the pillars, which significantly limits the throughput or efficiency in obtaining a large amount of experimental data^13^. In skeletal muscle cells, a recent study also suggested that the contractile force of engineered three-dimensional (3D) skeletal muscle tissue or thin muscular film could be calculated from the amount of deflection of a cantilever or elastic pillars or curvature of muscular thin film^14,15^. In particular, the engineered muscle tissue showed increases in alignment and maturation of myotubes compared with two-dimensional (2D) monolayer cultured myotubes, which is a potential alternative model for tissue^16^. Although this tissue can be used to evaluate changes in contractile force associated with muscle atrophy and hypertrophy^17,18^, these methods also require a complex tissue preparation procedure for attachment to the pillars, with low throughput, which is not suitable for drug screening. A simple and high-throughput assay system is typically established with conventional 2D cultured cells at the early stage of extensive screening to discover novel effective drugs, while a cell-based drug screening system assessing muscle strength as an indicator has not been developed to date. We therefore previously focused on developing an evaluation method for cellular traction force generated in epithelial cells undergoing collective migration, which looks at the specific deformation of silicone gel substrate (hereafter called the “substrate”)^19,20^. In this method, wrinkles on the substrate generated by cell migration or adhesion are acquired only with a standard optical microscope used in a routine manner. Since the length of wrinkles on the substrate is correlated with the magnitude of force exerted by the cells, the length of the wrinkles can be used to evaluate the cell-generated force^20,21^. In fact, with this assay it is possible to effectively and sensitively detect changes in cell traction force due to factors including drugs and gene mutations/knockdown/overexpression^22–26^. Though this system was originally developed to measure static adhesion forces or slowly varying forces during the migration of fibroblast and epithelial cells, we hypothesized that this system can also be applied to measure an acutely varying force like myotube contraction.

This study aims to establish a system to effectively and sensitively evaluate the relative change in force-related parameters in myotubes by measuring the length of wrinkles formed on the substrate. We confirmed that the contractile force, as indicated by a force index, was decreased in atrophic myotubes and increased in hypertrophic myotubes. Changes in myotube contractile force did not necessarily correspond to changes in myotube diameter or MHC protein expression; the new index could assess the phenotypes of myotubes more sensitively than existing parameters. These results indicate that this system can be used for the highly efficient assessment of the contractile force-related state of myotubes based on the change in substrate deformation. Given that there is currently no method for extensive drug screening using muscle strength as an indicator, which is highly anticipated in the field of muscle disease, our proposed method should be a novel and powerful tool for testing a number of candidate drugs to improve muscle weakness due to muscle-related diseases including muscle atrophy.

## Results

### Visualization of muscle contractile force as wrinkles formed on the substrate

**Figure 1:**
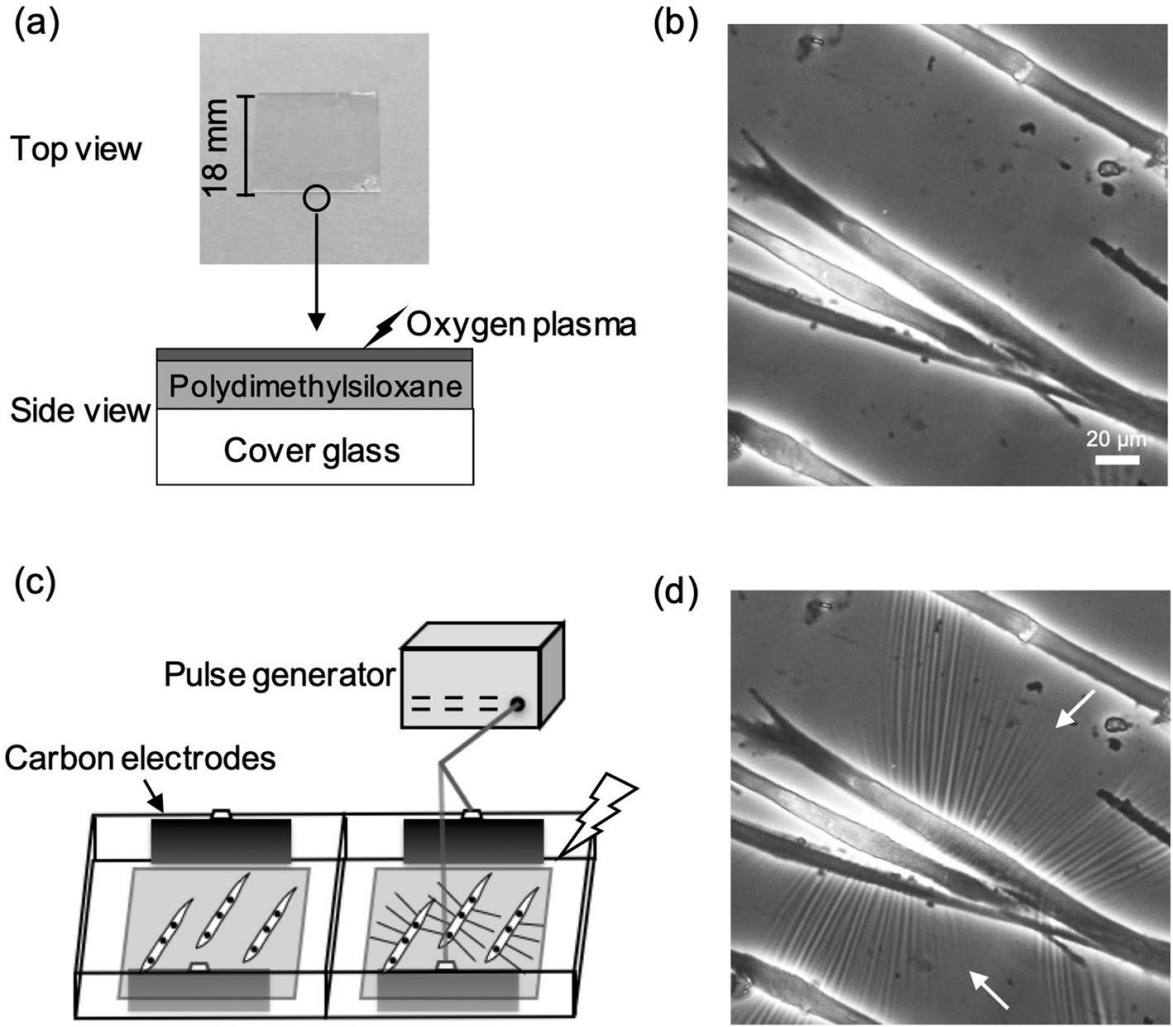
Wrinkles on the substrate are generated by contraction of the myotube. (**a**) The substrate used in this study and its schematics viewed from the side. The substrate is coated with a silicone gel (polydimethylsiloxane) on an 18 mm × 18 mm cover glass, and surface of the gel is treated by oxygen plasma. (**b**) Myotubes at day 5 of differentiation cultured on the substrate. Scale bar is 20 µm. (**c**) The 2-well chamber was connected to an electrical pulse generator with a pair of carbon electrodes for each well. The myotubes on the substrate were contracted with electrical pulses of 20 mA current, 20 ms duration, 980 ms interval, at 1 Hz (see Supplementary Movie 1). (**d**) The contraction of the myotubes in response to the electrical pulse generated wrinkles on the substrate. White arrows indicate wrinkles on the substrate.

The substrate was prepared by hardening the surface of a silicone gel (polydimethylsiloxane) coated on a cover glass by oxygen plasma treatment (Fig. 1a). Myotubes obtained from myoblasts seeded on the substrate were well-differentiated on day 5 (Fig. 1b). The myotubes exhibited repeated contraction in response to stimulation by electrical pulses from a pulse generator (20 mA, 1 Hz, 20 ms, and 980 ms intervals) without detaching from the substrate (Fig. 1c). This was immediately followed by the formation of wrinkles on the substrate (Fig. 1d and Supplementary Movie 1). The wrinkles were seen to form rhythmically in time with myotube contraction, indicating that the contractile force can be directly visualized as wrinkles on the substrate.

### Evaluation of the relationship between the applied mechanical force and length of wrinkles on the substrate

**Figure 2:**
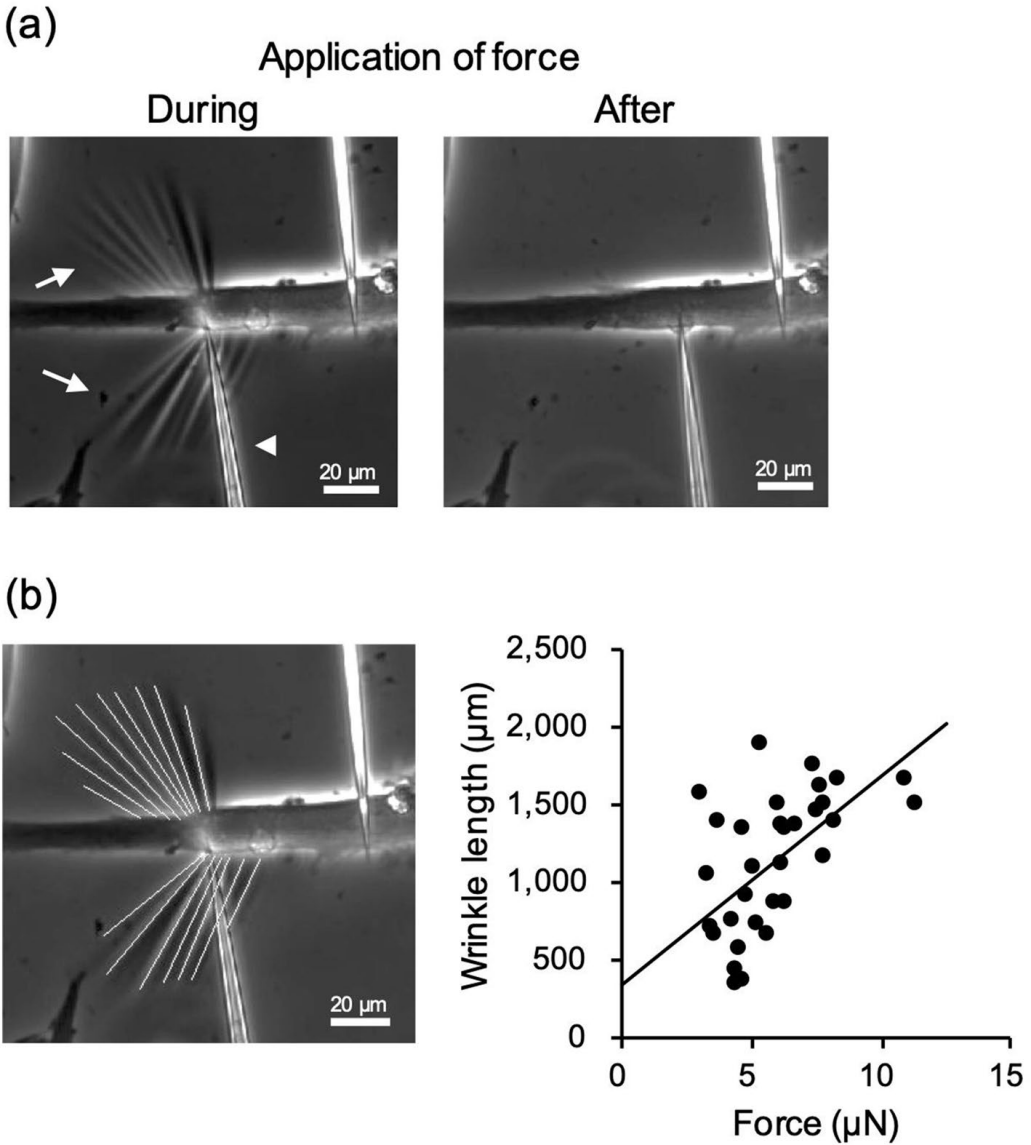
Correlation between the applied mechanical force and length of the wrinkles. The correlation was evaluated between the applied mechanical force and the total length of wrinkles formed on the substrate. Myotubes on the 5th day of differentiation on the substrate were fixed with 1% glutaraldehyde; a force was then applied with a flexible glass needle (see Supplementary Movie 2). (**a**) Fixed myotubes during and after the application of mechanical force with a needle (white arrowhead). The applied force formed wrinkles (white arrow) on the substrate. Scale bar is 20 µm. (**b**) Relationship between the force applied (µN) to the myotube and the total length of wrinkles (µm). The length of the wrinkles was measured using ImageJ-Fiji. The extracted wrinkles are shown as white lines. Scale bar is 20 µm. The force was calculated as the product of the bending stiffness and deflection of the needle (see Materials and Methods). Linear regression is shown, using the least-squares method (N = 32, where N represents the number of myotubes, r = 0.64, *P* < 0.05). The length of wrinkles is correlated with the applied force on the substrate.

The length of wrinkles on a substrate correlates with the magnitude of applied force, as reported in previous studies^20,21^. To assess the relationship between the wrinkle length and the applied force on the substrate in the present system, a mechanical force was applied to myotubes fixed with 1% glutaraldehyde. We used a flexible glass needle whose bending stiffness was measured separately in advance (Supplementary Movie 2). As shown in Fig. 2a, wrinkles were formed on the substrate during the application of the external force; they also disappeared when the needle was moved upward, releasing the force on the cell. The total length of the wrinkles and the applied force (described in the Materials and Methods section) were then simultaneously plotted. We observed a linear and significant positive relationship between the applied force and the length of wrinkles (r = 0.64, *P* < 0.05) (Fig. 2b), suggesting that the total length of wrinkles can be used to evaluate the contractile force generated by the myotubes.

### Development of an algorithm to evaluate the contractile force of myotubes

**Figure 3:**
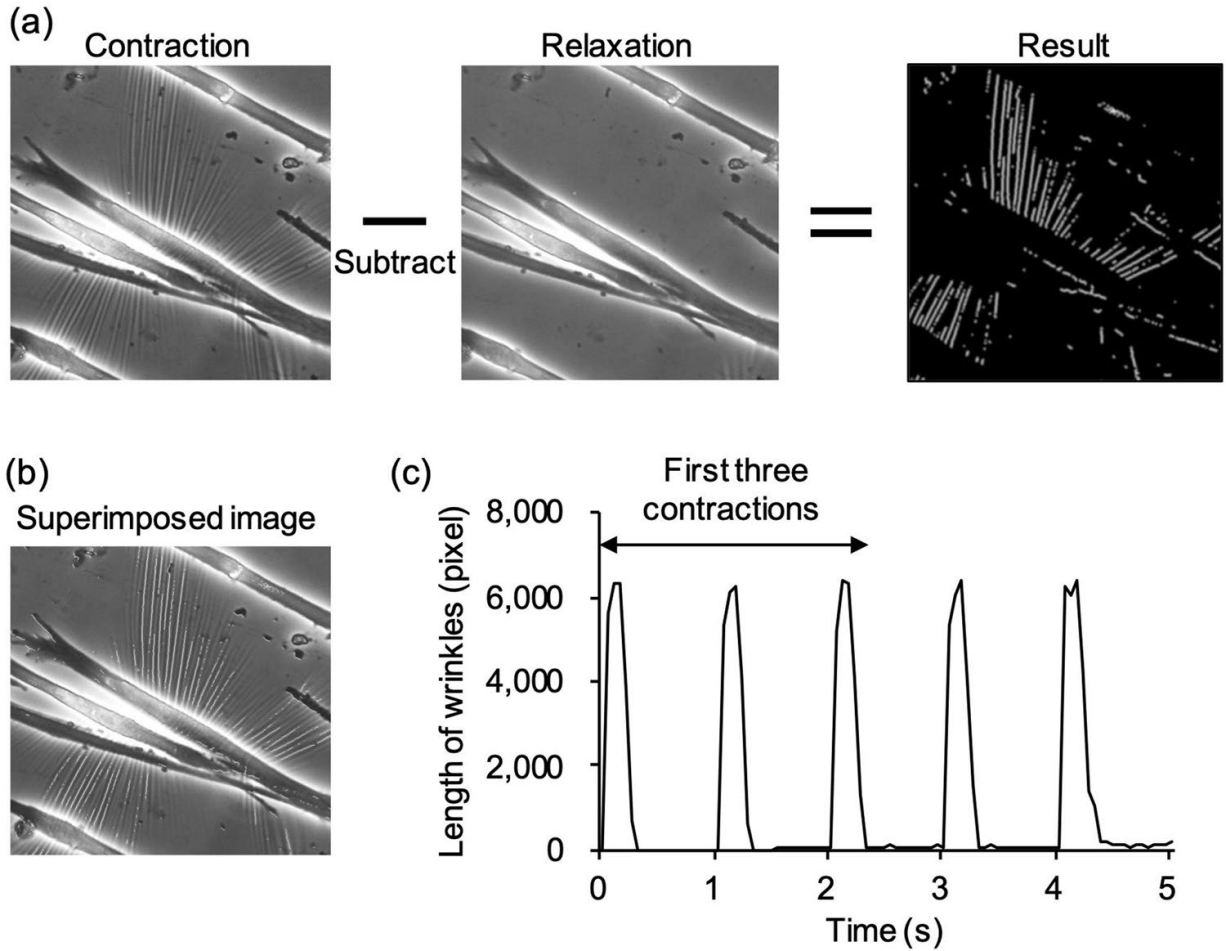
Evaluation of contractile force from wrinkles formed on the substrate. (**a**) How wrinkles on the substrate are extracted. The source movie was divided into frames. The wrinkles were extracted from the difference between images of contracted myotubes and relaxed myotubes. The difference image is binarized so the wrinkles appear as brighter lines. (**b**) Extracted wrinkles superimposed on the source movie (see Supplementary Movie 3), shown as white lines. (**c**) The length of wrinkles was found in pixels using ImageJ-Fiji. The total length of wrinkles generated by the first three contractions of myotubes was defined as a “force index”.

To evaluate the contractile force of myotubes using the length of wrinkles, we developed an algorithm that automatically analyzes the wrinkles formed on the substrate. Figure 3a is a representative image showing the lines extracted from wrinkles generated by myotube contraction. The myotube contraction movies were converted to image sequences, or frames. In the frames where the myotube contracts, the formed wrinkles appear as brighter lines. Thus, the wrinkles were extracted by taking the difference between frames corresponding to the contracted and relaxed states. Details are given in the Materials and Methods section. The result consists of white lines left where the wrinkles are. Merging these “extracted” wrinkles and the original image, as shown in Fig. 3b, shows a close match, showing that this algorithm could effectively extract the location of wrinkles generated by contraction. The total length of wrinkles was measured for each time-series image in pixels. This quantity was observed to be synchronized with muscle contraction induced by electrical stimulation input. We calculated the total length of the wrinkles generated by the first three contractions of myotubes (Fig. 3c) and defined this as a “force index” characterizing the contraction force.

**Figure 4:**
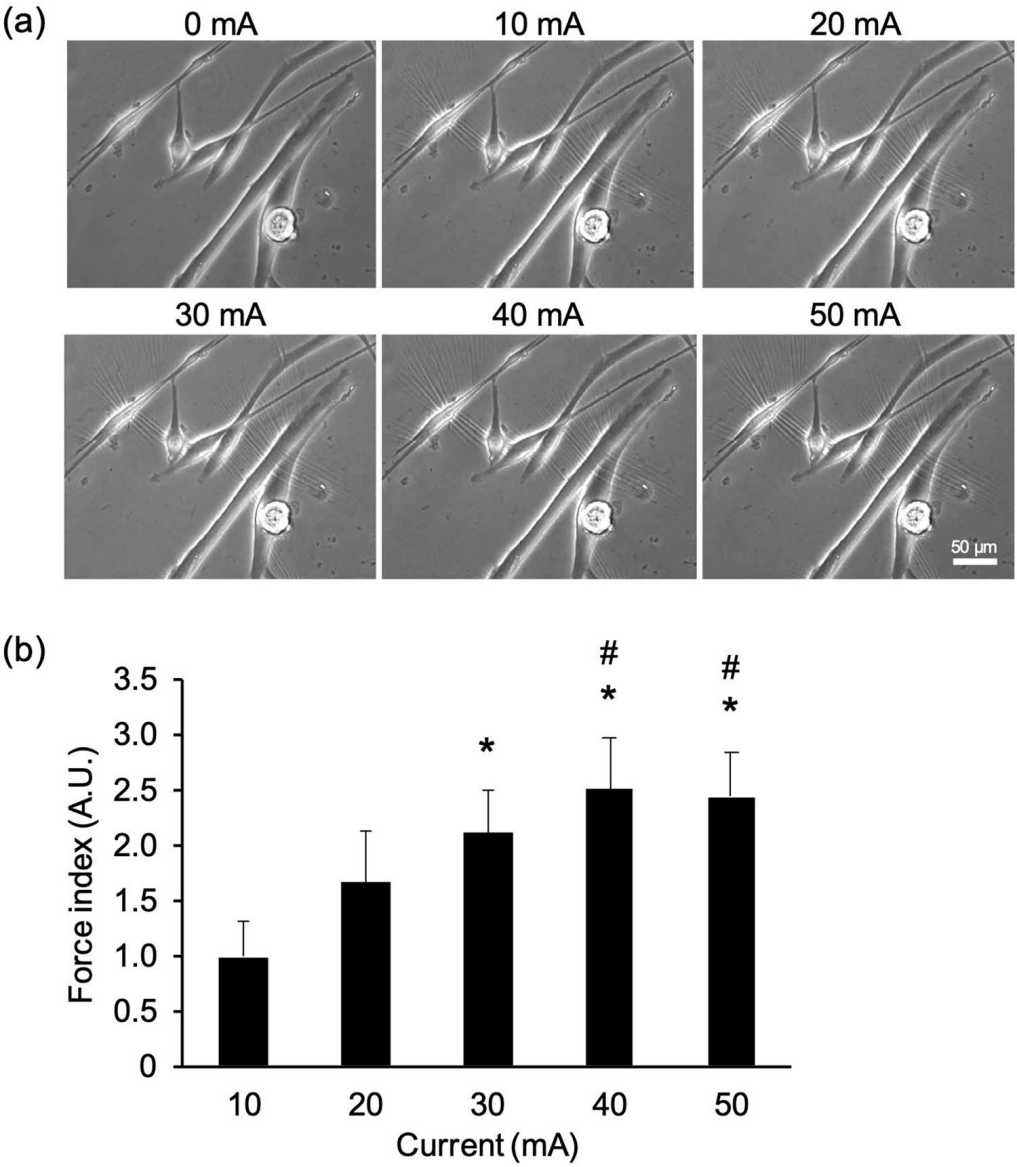
Contractile force of myotubes with different intensities of electrical current. The contractile force of myotubes was evaluated from the total length of wrinkles formed on the substrate. Myotubes on the 5th day of differentiation were stimulated with electric pulses of 0, 10, 20, 30, 40, and 50 mA current at 1 Hz (20 ms duration, 980 ms intervals). (**a**) Wrinkles on the substrate are generated by the contraction of myotubes in response to pulses with different electrical current. Scale bar is 50 µm. (**b**) Evaluation of contractile force of myotubes with different intensities of electrical current. The contractile force in response to each current was expressed by the force index (see Materials and Methods). The force index increased significantly with current intensity. N = 4 (N represents an independent experiment; each “N” shows the average value from 12 areas on the same substrate). Data are shown as mean ± S.E.M. Significance is indicated using a one-way ANOVA test, *P* < 0.01, followed by a Bonferroni post hoc test (*P  < 0.05 vs. 10 mA, #*P*  < 0.05 vs. 20 mA).

To assess whether the force index reflects how the wrinkles vary with the intensity of the electrical stimulation, myotubes were stimulated by five different intensities of electrical current at 1 Hz, 20 ms duration, and 980 ms interval. With an increased current, the length of wrinkles on the substrate was apparently increased, as shown in Fig. 4a and Supplementary Fig. 1a. The quantified force index also increased in response to the intensity of the current (Fig. 4b). From a material perspective, our silicone materials behave elastically, and they allow full responses to rapid myotube contraction of at least 1 Hz^27^. This reinforces the assertion that the algorithm successfully extracts a measure of twitch contractile force from the length of wrinkles on the substrate. We also analyzed the time to peak twitch force (TPT) and half relaxation time (HRT) of the contractile properties in myotube twitch contractions. However, TPT and HRT were unchanged at all current intensities (Supplementary Fig. 1b and c), which is supported by previous reports describing that TPT and HRT vary less than force index^28^.

To evaluate whether the wrinkles generated by myotube contraction also reflect changes in the tetanic contraction force in myotubes, the myotubes were stimulated by five different intensities of electrical current at 100 Hz consisting of 2 ms duration for 3 s per cycle. The myotubes showed sustained tetanic contraction at all currents. Supplementary Movie 4 shows the wrinkles generated on the substrate by tetanic contraction. With increasing current, the length of wrinkles on the substrate was increased, as shown in Supplementary Fig. 1d. The calculated force index tended to increase, but this failed to be statistically significant in an electrical current-dependent manner (Supplementary Fig. 1e).

From these results, we selected a 20 mA electrical pulse at 1 Hz as a stimulus for subsequent experiments that allowed a sufficient increase and decrease in the force index, calculated from the total length of wrinkles generated by the contraction of myotubes under different conditions.

### Assessment of atrophy and hypertrophy in myotubes

**Figure 5:**
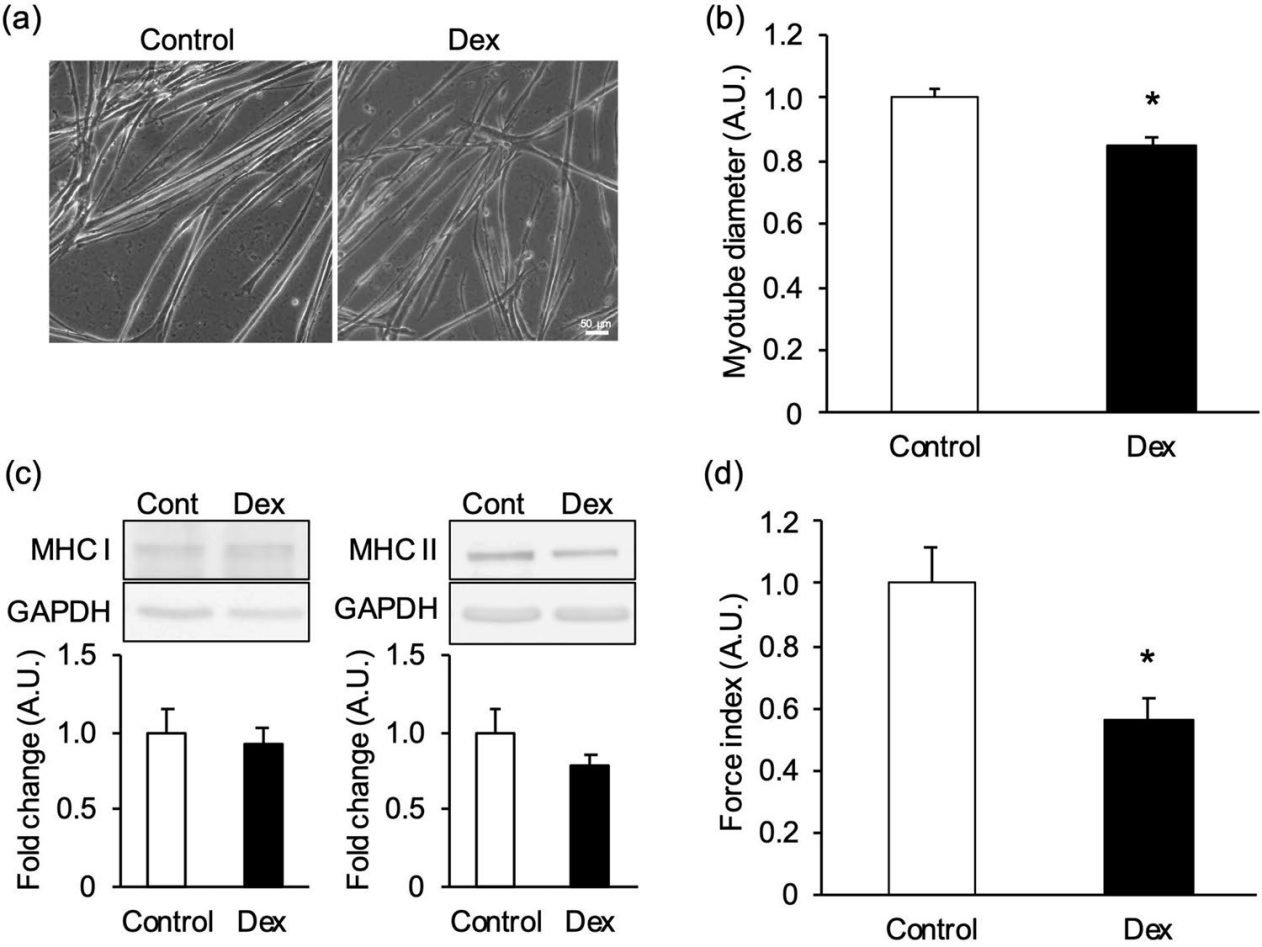
Evaluation of muscle mass and contractile force in myotubes treated with dexamethasone. Dexamethasone (Dex)- induced atrophic myotubes were evaluated by their diameter, myosin heavy chain (MHC) expression levels, and “force index,” an index of contractile force. Myotubes were treated with 100 mM dexamethasone in the differentiation medium on the 5th day of differentiation and cultured for 48 h. Control cells were treated with 0.1% (v/v) DMSO (Control). (**a**) Control- and dexamethasone- treated myotubes cultured on the substrate. Scale bar is 50 µm. (**b**) Diameter of dexamethasone- treated myotubes. The myotube diameter treated with dexamethasone was lower than that of the control. N = 10 (N represents an independent experiment; each “N” shows the average value from all myotubes of five random fields on the same substrate). (**c**) Representative blots of MHC I, MHC II, and glyceraldehyde-3-phosphate dehydrogenase (GAPDH), and quantification of the proteins on western blots. No significant changes in MHC I or II were observed between Control and dexamethasone. Blots are cropped original images of MHC I, MHC II, and GAPDH. Original images are presented in Supplementary Fig. 3. N = 10. (**d**) Force index as an index of contractile force. The myotubes were stimulated by an electrical pulse (20 mA, 20 ms duration, 980 ms intervals, 1 Hz). The force index for dexamethasone- treated myotubes was significantly lower than that of the control. N = 10 (N represents an independent experiment; each “N” shows the average value from 12 areas on the same substrate). Data are shown as mean ± S.E.M., *P < 0.05, vs. control (Student’s t-test).

We next examined whether this assay system can detect changes in contractile force in atrophic and hypertrophic myotubes. To induce myotube atrophy, myotubes were treated with 100 mM dexamethasone (Fig. 5a). In general, atrophy evaluation in myotubes is undertaken by measuring the myotube diameter and protein expression level of myosin heavy chain I/II (MHC I/II). Since the degradation response is different between MHC I and MHC II depending on the type of atrophy e.g. aging or disuse atrophy, the levels of expression of MHC I and MHC II were evaluated separately in the study. Treatment with dexamethasone induced a decrease in myotube diameter by 15.3 ± 2.8% compared with that of control cells (Fig. 5b). On the other hand, MHC I and II protein expression levels were unchanged (Fig. 5c). The wrinkles formed on the substrate were observed for control- and dexamethasone-treated myotubes (Supplementary Fig. 2a). The force index for dexamethasone-treated myotubes was significantly decreased, by 43.6 ± 6.9% (Fig. 5d).

**Figure 6:**
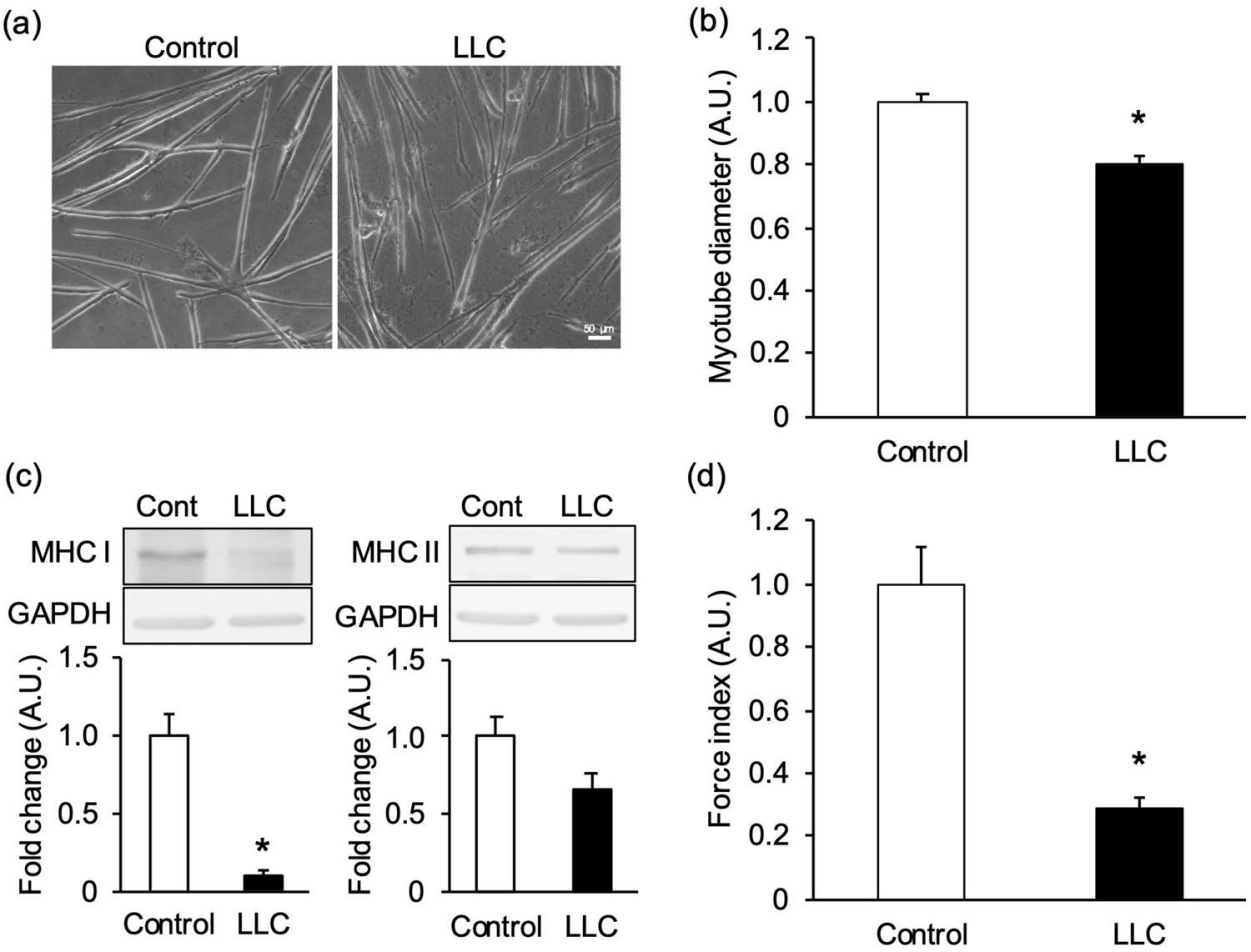
Evaluation of muscle mass and contractile force in myotubes treated with cancer cell-conditioned medium. Cancer cachexia-induced atrophic myotubes were evaluated by their diameter, myosin heavy chain (MHC) expression levels, and “force index,” an index of contractile force. Myotubes were treated with a differentiation medium containing 50% (v/v) of the conditioned medium of Lewis lung carcinoma (LLC) on the 3rd day of differentiation and cultured for 4 days. Control cells were treated with a differentiation medium containing 50% (v/v) of the conditioned medium of myotubes (Control). (**a**) Control- and LLC- conditioned medium treated myotubes cultured on the substrate. Scale bar is 50 µm. (**b**) Diameter of LLC- conditioned medium treated myotubes. The myotube diameter treated LLC- conditioned medium was lower than that of the control. N = 6 (N represents an independent experiment; each “N” shows the average value from all myotubes of 5 random fields on the same substrate). (**c**) Representative blots of MHC I, MHC II, and Glyceraldehyde-3-phosphate dehydrogenase (GAPDH), and quantification of the proteins on western blots. The MHC I protein expression level of LLC- conditioned medium treated myotubes was lower than that of the control. However, no significant change in MHC II was observed. Blots are cropped original images of MHC I, MHC II, and GAPDH. Original images are presented in Supplementary Fig. 4. N = 6. (**d**) Force index as an index of contractile force. The myotubes were stimulated by an electrical pulse (20 mA, 20 ms duration, 980 ms intervals, 1 Hz). The force index of LLC- conditioned medium treated myotubes was significantly lower than that of control. N = 6 (N represents an independent experiment; each “N” shows the average value from 12 areas on the same substrate). Data are shown as mean ± S.E.M., *P < 0.05, vs. control (student’s t-test).

We next considered atrophy in a model for cancer cachexia. Myotubes treated with Lewis Lung Carcinoma (LLC) conditioned medium (see Materials and Methods) were found to suffer severe atrophy (Fig. 6a). The myotube diameter treated with LLC conditioned medium was significantly decreased, by 19.5 ± 2.1%, compared with that of control cells (Fig. 6b). The MHC I protein expression level in LLC conditioned medium treated myotubes was significantly decreased (Fig. 6c), whereas MHC II protein expression levels tended to decrease but failed to be statistically significant. The wrinkles formed on the substrate were observed for control- and LLC conditioned medium-treated myotubes (Supplementary Fig. 2b). The force index for LLC conditioned medium-treated myotubes was significantly decreased, by 71.0 ± 3.1% (Fig. 6d). These results suggest that the force index is more sensitive to detecting muscle atrophy than myotube diameter or MHC protein expression.

**Figure 7:**
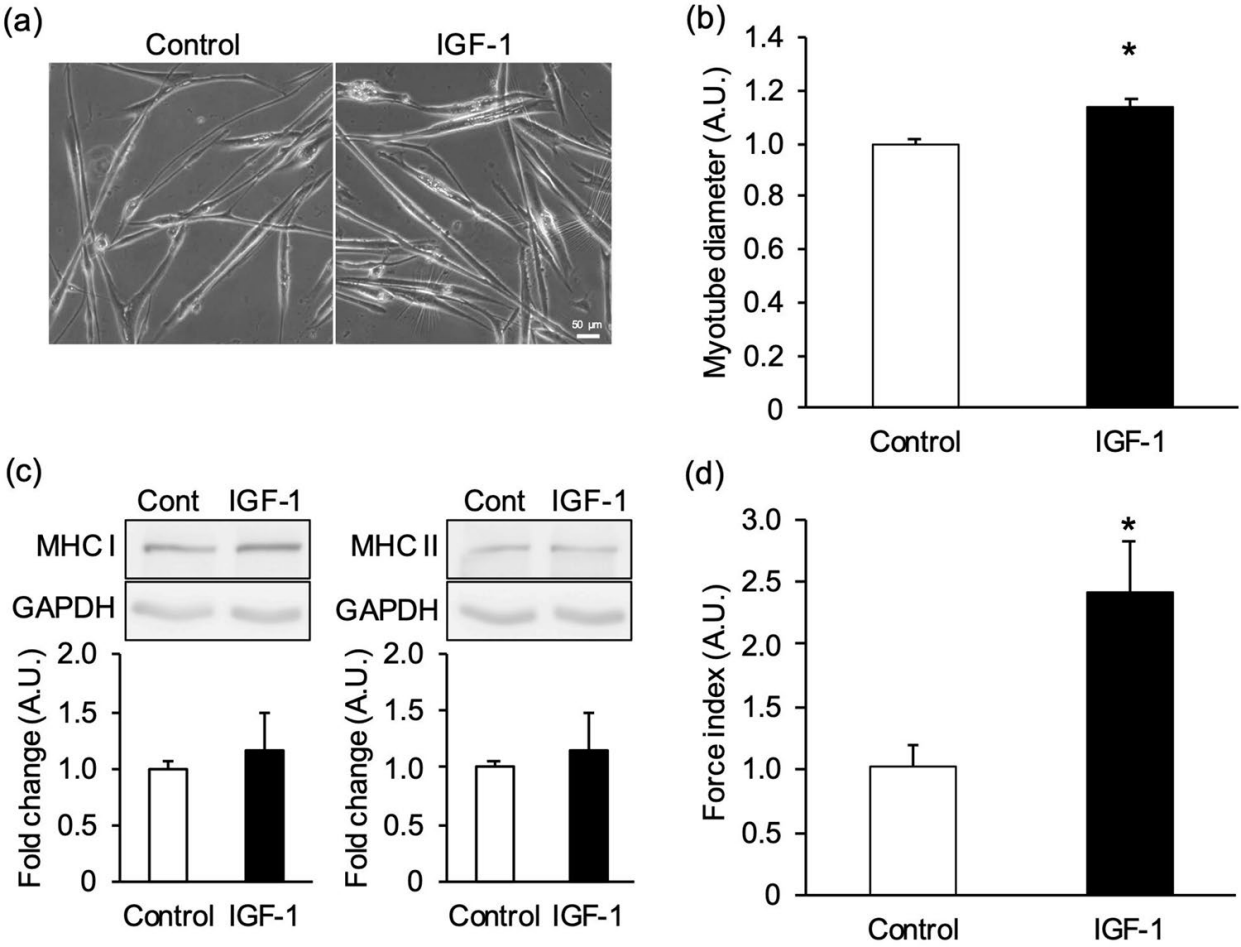
Evaluation of muscle mass and contractile force in myotubes treated with IGF-1. Insulin-like growth factor-1 (IGF-1) induced hypertrophic myotubes were evaluated by their diameter, myosin heavy chain (MHC) expression levels, and “force index,” an index of contractile force. Myotubes were treated with 100 ng/mL IGF-1 in the differentiation medium from the start of differentiation for 7 days (IGF-1). Control cells were treated with 0.1% (v/v) 10 mM Tris–HCl in the differentiation medium (Control). (**a**) Control- and IGF-1-treated myotubes cultured on the substrate. Scale bar is 50 µm. (**b**) The diameter of IGF-1 treated myotubes. The myotube diameter treated with IGF-1 was higher than that of the control. N = 5 (N represents an independent experiment; each “N” shows the average value from all myotubes of 5 random fields on the same substrate). (**c**) Representative blots of MHC I, MHC II, and glyceraldehyde-3-phosphate dehydrogenase (GAPDH), and quantification of the proteins on western blots. No significant changes in MHC I or II were observed between the control and IGF-1. Blots are cropped original images of MHC I, MHC II, and GAPDH. Original images are presented in Supplementary Fig. 5. N = 8. (**d**) Force index as an index of contractile force. The myotubes were stimulated by an electrical pulse (20 mA, 20 ms duration, 980 ms intervals, 1 Hz). The force index of IGF-1-treated myotubes was significantly higher than that of the control. N = 5 (N represents an independent experiment; each “N” shows the average value from 12 areas on the same substrate). Data are shown as mean ± S.E.M., *P < 0.05, vs control (Student’s t-test).

Next, we assessed whether this system could detect intensified contraction with hypertrophic myotubes. Myotubes were treated with 100 ng/mL insulin-like growth factor-1 (IGF-1), which promotes muscle protein synthesis (Fig. 7a). The myotube diameter treated with IGF-1 was significantly increased, by 14.8 ± 3.1% compared with that of control cells (Fig. 7b). In contrast, MHC I and MHC II protein expression levels were unchanged (Fig. 7c). The wrinkles formed on the substrate were observed for control- and IGF-1-treated myotubes (Supplementary Fig. 2c). The force index in IGF-1 treated myotubes was significantly increased, by 140.4 ± 41.6% (Fig. 7d). These results suggest that the force index can detect the phenotype of myotubes with high sensitivity, not only in muscle atrophy but also hypertrophy.

## Discussion

In this study, we established a simple system to effectively and sensitively evaluate the contractile force of myotubes. A deformable silicone gel substrate can visualize the magnitude of the applied force as wrinkles on its surface. Our system, in which a controllable electric pulse generator^29,30^, is newly combined with a deformable substrate, allows us to evaluate the current-triggered contractile force of myotubes using a force index, found from the total length of the wrinkles formed on the substrate. The force index was significantly decreased in atrophic myotubes and increased in hypertrophic myotubes even under conditions with little or no change in the myotube diameter or MHC protein expression. This suggests that the force index can detect the change in the phenotypes of myotubes with high sensitivity. These results show that our new contractile force assay system is suitable for assessing the degree of muscle condition due to atrophy and hypertrophy, and useful for in vitro drug screening for improving muscle weakness.

Attempts to assess the traction forces exerted by epithelial cells and fibroblasts in vitro have been reported. Munevar et al. measured the traction forces of cultured cells using deformable gels embedded with fluorescent micro-beads^12^. In this method, the traction force of cells is typically detected using confocal fluorescence imaging, extracting the change in positions of the beads due to deformation of the gel. The magnitude and direction of cell-driven traction forces can be quantified from the distance the bead travel and the elastic modulus of the gel. Although this method can be used to measure the cell static force, tracking the beads by muscle contraction is difficult due to instantaneous changes in bead position. Instead, our system simply uses a phase-contrast microscope with a high frame rate camera followed by analysis of the recorded movies. Since the wrinkles formed on the substrate in the recorded movies are automatically analyzed to determine the force index, technical expertise and experience are not required, making it appropriate for wide use.

We used two different atrophy models to determine whether this system can detect any type of atrophy, since the underlying molecular mechanism of each atrophy type differs depending on the factors that induced it. Glucocorticoids are elevated in sepsis, cachexia, starvation, metabolic acidosis, and severe insulinopenia, and induces the atrophy of type II muscle fibers mainly expressing MHC II^31^. Dexamethasone, a glucocorticoid receptor agonist, induces gene expression of forkhead box O (FoxO), kruppel-like factor 15 (KLF15), Atrogin-1, and muscle RING finger 1 (MuRF-1), resulting in protein breakdown. Dexamethasone also inhibits the mammalian target of rapamycin (mTOR) pathway for protein synthesis^32–34^. On the other hand, cancer cachexia is induced by cancer cell-secreted factors such as TNF-α and IL-6, and these induce the atrophy of both type I muscle fibers mainly expressing MHC I and type II muscle fibers^35^. IL-6 and TNF-α activate JAK/STAT pathways and nuclear factor-kappa B (NF-κB), respectively, induce ubiquitin–proteasome proteolysis and autophagy^36–39^. Although the atrophic mechanisms and fiber types behind each atrophy are different, it all ends up in reducing muscle strength. Therefore, our results provide important insight into the ability of the new system to perform drug screening for the two atrophic types.

In general, muscle strength and mass are highly correlated; it is thought that increasing muscle mass directly leads to the recovery of muscle strength. However, maintaining or increasing muscle mass in older adults does not prevent aging-derived decline in muscle strength^40^. Furthermore, in clinical trials, drugs for muscle atrophy targeting the myostatin-activin signal pathway have been reported to increase muscle mass but not significantly restore muscle strength or physical function^9,10^. Since it has been suggested that it is low muscle strength that is related to limited physical function rather than decreased muscle mass^4^, it is critical to evaluate whether loss of muscle strength is prevented or restored when developing therapeutic drugs for muscle atrophy. The relative change in the force index was greater than that seen in the myotube diameter or MHC protein expression due to atrophy or hypertrophy, suggesting that the force index detects changes in muscle function more directly and hence more sensitively. Therefore, the system we established in this study may be more suitable than existing methods for screening therapeutic drugs to prevent or restore low muscle strength due to muscle atrophy.

Our aim here is to evaluate the relative change in force-related parameters, not measure the absolute value of cellular force. We showed a positive correlation between the force and wrinkle length (Fig. 2), and cell-induced wrinkles were also clearly lengthened in response to the current-induced contraction of the myotubes (Fig. 4). Here, we decided to employ 20 mA, which the relative change between the current and force index are in a nearly linear relationship. Our results showed that our system was able to evaluate the relative change in contraction force between atrophic and hypertrophic myotubes. Thus, from the standpoint of “assay” that analyzes the relative change, it is sufficient to detect the change in length of force-induced wrinkles as an indicator of the contractile force of myotubes. As described in the introduction, TFM^12^, a cantilever^14^, and elastic pillars^14,16–18^ are also valuable for determining the absolute value of cellular force, although the procedure for device creation and muscle tissue preparation is complex, and there are significant limitations in throughput and efficiency for obtaining a large amount of experimental data. Given that there is currently no method available for extensive drug screening using muscle strength as an indicator, which is highly anticipated in the field of muscle diseases, our proposed method is clearly technically novel and has the potential to test a number of candidate drugs to improve muscle-related diseases.

A major limitation of the system is the lack of correction for myotube alignment and the number of myotubes on the substrates. As shown in Supplementary Fig. 6, the number of myotubes and their alignment are different in each image. Thus, artificial control of myotube density and alignment using, for example, micropatterning techniques will be a subject of future studies, although there is currently no appropriate correction method that compensates for variations in the number and alignment of myotubes. To minimize any potential bias, images containing one to four contracting cells per field of view were randomly captured in twelve different areas on the substrate, and the force index was averaged. Although biases among substrates may not be fully excluded using this method, our data clearly showed that the force index changes significantly with muscle atrophy as well as hypertrophy, suggesting that the results can be widely used to evaluate contractile force without employing complex analytical tools.

In summary, we established a simple system to effectively and sensitively evaluate relative changes in contractile force of cultured myotubes by combining a deformable silicone gel substrate with an electric stimulation system. This system is sufficiently sensitive to assess the myotube condition due to atrophic and hypertrophic myotubes using a force index, and promises to be useful in the development and screening of drugs for the preventing and/or improving muscle weakness.

## Materials and methods

### Animals

C57BL/6 N adult mice (8–12 weeks old, male) were housed in a cage in a temperature-controlled room at 24˚C with 12 h (4:00 a.m.–4:00 p.m.) light–dark cycle. All animal experiments were approved by the Animal Care Committee of Tokyo Metropolitan University (approval number A28-7, A29-14, A30-8, and A31-20) and performed in accordance with relevant guidelines and regulations. This study was conducted in compliance with the ARRIVE guidelines.

### Cell culture

Extensor digitorum longus (EDL) muscles were isolated from C57BL/6 N mice. Satellite cells were prepared as described previously^41^ with some modifications. In brief, EDL was digested with 0.2% type I collagenase (Worthington, Lakewood, NJ, U.S.A) in Dulbecco's Modified Eagle Medium (DMEM) high glucose GlutaMAX (Thermo Fisher Scientific, Waltham, MA, U.S.A) for 2 h at 37˚C. Isolated myofibers were collected and digested by Accutase (Innovative Cell Technologies, San Diego, CA, U.S.A) for 10 min at 37˚C. Digested myofibers were then seeded onto Matrigel (BD Biosciences, Franklin Lakes, NJ, USA) coated 150 mm culture dishes in a growth medium consisting of DMEM, no glucose (Thermo Fisher Scientific) supplemented with 30% FBS (511–98,175, FUJIFILM Wako Pure Corporation Chemical, Osaka, Japan), 1% GlutaMAX (Thermo Fisher Scientific), 1% chicken embryo extract (US Biological, Marblehead, MA, U.S.A), 10 ng/ml bFGF, and 1% anti-biotic (Thermo Fisher Scientific) at 37˚C with 5% CO_2_ to form myoblasts on culture dishes^42^. Myoblasts were re-seeded at a density of 3.0 × 10^4^ cells per well onto Matrigel-coated silicone substrates that attached to the bottom of 2-well chambers (Thermo Fisher Scientific) (see “contractile force assay” part). 1 day after seeding, the medium was switched to a differentiation medium consisting of DMEM high glucose GlutaMAX supplemented with 5% horse serum (16,050,130, Thermo Fisher Scientific) and 1% anti-biotic. Half the amount of medium was replaced every day. Myotubes were used for contractile force assays 5 or 7 days after differentiation.

LLC cells (kind a gift from Dr. Hata, Y. Tokyo Medical and Dental University) were cultured in a growth medium consisting of DMEM high glucose supplemented with 10% horse serum (16,050,130, Thermo Fisher Scientific) and 1% anti-biotic. The medium was changed every 2 days to maintain the cells.

### Atrophic and hypertrophic myotubes

To produce the dexamethasone-induced atrophic myotubes, myotubes were treated with 100 mM dexamethasone (Sigma Aldrich, MO, U.S.A) in the differentiation medium on the 5th day of differentiation and cultured for 48 h. Control cells were treated with 0.1% (v/v) dimethyl sulfoxide (DMSO; Sigma Aldrich) in the differentiation medium. To produce cancer cachexia-induced atrophic myotubes, LLC and myoblasts were cultured separately in the differentiation medium for 48 h at 2.5 × 10^6^ cells/100 mm plate; the conditioned medium were then collected. The conditioned medium from LLC was mixed with fresh differentiation medium at 50% (v/v) and used as the cachexia medium. The conditioned medium from myotubes was mixed with fresh differentiation medium at 50% (v/v) and used as a control medium^43^. Myotubes were treated with the cancer cachexia medium on the 3rd day of differentiation and cultured for 4 days. Control cells were treated with the control medium. To produce the IGF-1 induced hypertrophic myotubes, myotubes were treated with 100 ng/mL IGF-1 (Sigma Aldrich) in the differentiation medium from the start of differentiation for 7 days. Control cells were treated with 0.1% (v/v) 10 mM Tris–HCl in the differentiation medium.

### Contractile force assay

The contractile force of myotubes was evaluated using a deformable silicone substrate technique^19,20^ with some modifications. Parts A and B of CY 52–276 (Dow Corning TORAY, Tokyo, Japan) were mixed at a mass ratio of 1.4:1 to form a silicone gel. The gel was coated on an 18 mm × 18 mm cover glass (Matsunami Glass, Osaka, Japan) using a spin coater (K-359S1; Kyowariken, Tokyo, Japan) at 500 rpm for 10 s and 1500 rpm for 30 s and baked at 60˚C for 20 h in a drying oven to cure the gel (Tokyo Garasu Kikai, Tokyo, Japan). The gel surface was then treated with oxygen plasma (2 mA, 100 V, 20 Pa, 1 min) (SEDE-GE; Meiwafosis, Tokyo, Japan). The substrate was attached to the bottom of 2-well chambers and coated with Matrigel for 30 min before cell seeding. Myoblasts were seeded on the substrate and allowed to differentiate for 5 or 7 days. The medium was switched to a differentiation medium supplemented with 10 mM HEPES (Nacalai tesque, Kyoto, Japan) immediately before the myotube contraction. The 2-well chamber was connected to a carbon electrode (Uchida Denshi, Tokyo, Japan) and an electrical pulse generator (Uchida Denshi). Myotubes were stimulated with electric pulses with 20 mA current at 1 Hz, with each cycle consisting of a 20 ms duration followed by a 980 ms interval. Wrinkle formation was observed under a phase-contrast microscope (ECLIPSE Ti; Nikon, Tokyo, Japan) at 200 × magnification and was recorded as a movie over 12 areas on the same substrate, acquired at 16 frames per second.

### Assessment of the relationship between force and wrinkles

A certain magnitude of mechanical force was applied to the fixed myotubes using a flexible glass needle as described previously^20^. The needle was made from a glass rod using a glass electrode puller (P-1000; Sutter Instrument, CA, U.S.A). The tip of the needle was made to be approximately 5 µm in diameter. The bending stiffness of the needle was determined to be 465 nN/µm; this was measured from the displacement of the tip of the needle when a constant force was applied to the needle using an atomic force microscopy cantilever with a precisely calibrated stiffness (OMCL-TR400PB-1; Olympus, Tokyo, Japan). Myotubes on the 5th day of differentiation were fixed with 1% glutaraldehyde in PBS for 10 min at room temperature and washed with 30 mmol/L glycine in PBS. The needle was pricked into a fixed myotube using a micromanipulator (MWO-3; Narishige, Tokyo, Japan) to apply mechanical force, and observed under the phase-contrast microscope. The deflection of the needle was measured as the distance between the tips of the needles during and after the application of force. The force applied to the myotubes was calculated as the product of the bending stiffness (nN/µm) and the deflection (µm) of the needle. All data were subjected to outlier tests and the data excluding outliers were plotted graphically.

### Extraction of wrinkles generated by myotube contraction

The wrinkles generated by the contractile force of myotubes were automatically extracted from the phase-contrast movie using the envelopment algorithm in ImageJ-Fiji. Movies of the myotube contraction were converted into image sequences. In order to identify bright regions corresponding to the cell contours (thus, not the wrinkles), a grayscale morphological operation and binarization was carried out on a frame showing relaxed myotubes. The identified area was set to zero in the images. This process is also necessary to exclude some wrinkles formed in non-cell areas on the substrate due to attachment of non-cell materials. The wrinkles generated exclusively by the myotube contraction were then extracted by subtracting the relaxed image from images where the myotube was contracted, leaving signal only where there were wrinkles. Finally, the extracted wrinkles were skeletonized into line segments; wrinkles comprised of less than 20 pixels were removed as noise. The total length of the wrinkles within the image in pixels was plotted over time.

### Measurement of myotube diameter

Images of the myotubes were acquired at 100 × magnification before the contractile force assay. The myotube diameters were measured in all cells from 5 random fields of view per substrate using NIS-Elements (Nikon).

### Western blotting

Myotubes were washed with PBS and harvested with 200 µL of lysis buffer containing 50 mM Tris–HCl (pH 7.5), 5 mM sodium pyrophosphate tetrabasic, 1 mM ethylenediaminetetraacetic acid (pH 8.0), 1 mM sodium orthovanadate, 1% Nonidet P-40, 10 mM sodium fluoride, 150 mM sodium chloride, 10 mg/L leupeptin, 1 mM phenylmethylsulfonyl fluoride, 5 mg/mL aprotinin, 3 mM benzamidine, and 10 mM beta glycerophosphate. Harvested cells were sonicated and centrifuged at 13,000 X g for 15 min at 4˚C, and the supernatant was used for immunoblotting. The protein concentration of the supernatant was determined by the Bradford protein assay. Cell lysates were separated by 8–10% sodium dodecyl sulfate–polyacrylamide gel electrophoresis and transferred to polyvinylidene fluoride membranes. The membranes were cut according to the molecular weight and blocked with Tris Buffered Saline containing 0.1% Tween 20 and 5% non-fat dry milk. The membranes after blocking were incubated with the primary antibodies of myosin heavy chain I (MHC I; 1:1000, Sigma), myosin heavy chain II (MHC II; 1:1000, Sigma) or glyceraldehyde-3-phosphate dehydrogenase (GAPDH; 1:3000, Cell Signaling, MA, U.S.A) overnight at 4˚C, followed by incubation with horseradish peroxidase-conjugated secondary antibody (GE Healthcare, Buckinghamshire, UK) for 1 h at room temperature. Blots were used for detection with enhanced chemiluminescence (PerkinElmer, MA, U.S.A), analyzed with ImageQuant LAS 4000 mini (GE Healthcare) and quantified using ImageQuant TL (GE Healthcare).

### Statistics

Data are shown as the mean ± S.E.M. An unpaired Student’s t-test was performed to evaluate statistical differences between the two groups, and values of *P* < 0.05 were considered to be statistically significant. For multiple comparisons, data were analyzed using a one-way ANOVA followed by Bonferroni post hoc test, and values of *P* < 0.05 were considered to be statistically significant.

## Supplementary Information

Supplementary Information 1.

Supplementary Information 2.

Supplementary Information 3.

Supplementary Information 4.

Supplementary Video 1.

Supplementary Video 2.

Supplementary Video 3.

Supplementary Video 4.

## Data Availability

No datasets were generated or analyzed during the current study. All data generated or analyzed during this study are included in this published article and its Supplementary Information files.
