# Supplementary material for: Establishment of a system evaluating the contractile force of electrically stimulated myotubes from wrinkles formed on elastic substrate
Source: Sci Rep. 2022 Aug 15;12:13818. doi: 10.1038/s41598-022-17548-7 (PMC9378739; doi:10.1038/s41598-022-17548-7)
Supplement: Supplementary file 1 — Supplementary Information 1. [file 41598_2022_17548_MOESM1_ESM.pdf]

Supplementary Information

Establishment of a system evaluating the contractile force of electrically stimulated myotubes from wrinkles formed on elastic substrate

Hiroki Hamaguchi[1], Tsubasa S. Matsui[2], Shinji Deguchi[2], Yasuro Furuichi[1], Nobuharu L. Fujii[1] and Yasuko Manabe[1]*

[1]Department of Health Promotion Sciences, Graduate School of Human Health Sciences, Tokyo Metropolitan University, 1-1 Minami-Osawa, Hachioji, Tokyo, 192-0397, Japan

[2]Division of Bioengineering, Graduate School of Engineering Science, Osaka University, 1-3 Machikaneyama, Toyonaka, Osaka, 560-8531, Japan

*Corresponding author:

e-mail: ymanabe@tmu.ac.jp; Tel.: +81-42-677-2965; Fax: +81-42-677-2961

Materials and Methods

Evaluating the time to peak twitch force (TPT) and half relaxation time (HRT) of the myotube twitch contraction

Myotubes were stimulated with electric pulses of 10, 20, 30, 40, and 50 mA current at 1 Hz (20 ms duration, 980 ms intervals). Time to peak twitch force (TPT) and half relaxation time (HRT) were calculated from a graph plotting the total length of wrinkles over time. The TPT and HRT were calculated as previously described [1].

Evaluating the tetanic contractile force of myotubes

Myotubes were stimulated with the electric pulse of 10, 20, 30, 40, and 50 mA current at 100 Hz consisting of 2 ms duration for 3 s per cycle, as previously described [2]. The total length of the wrinkles generated by myotube contractions was extracted and the force index was calculated as described in materials and methods.

References

1. Hinds, S. Bian, W. Dennis, R. G. & Bursac, N. The role of extracellular matrix composition in structure and function of bioengineered skeletal muscle. *Biomaterials.* **32**, 3575-3583 (2011).
2. Sato, S. et al. A new in vitro muscle contraction model and its application for analysis of mTORC1 signaling in combination with contraction and beta-hydroxy-beta-methylbutyrate administration. *Biosci Biotechnol Biochem*. **83**, 1851-1857 (2019).

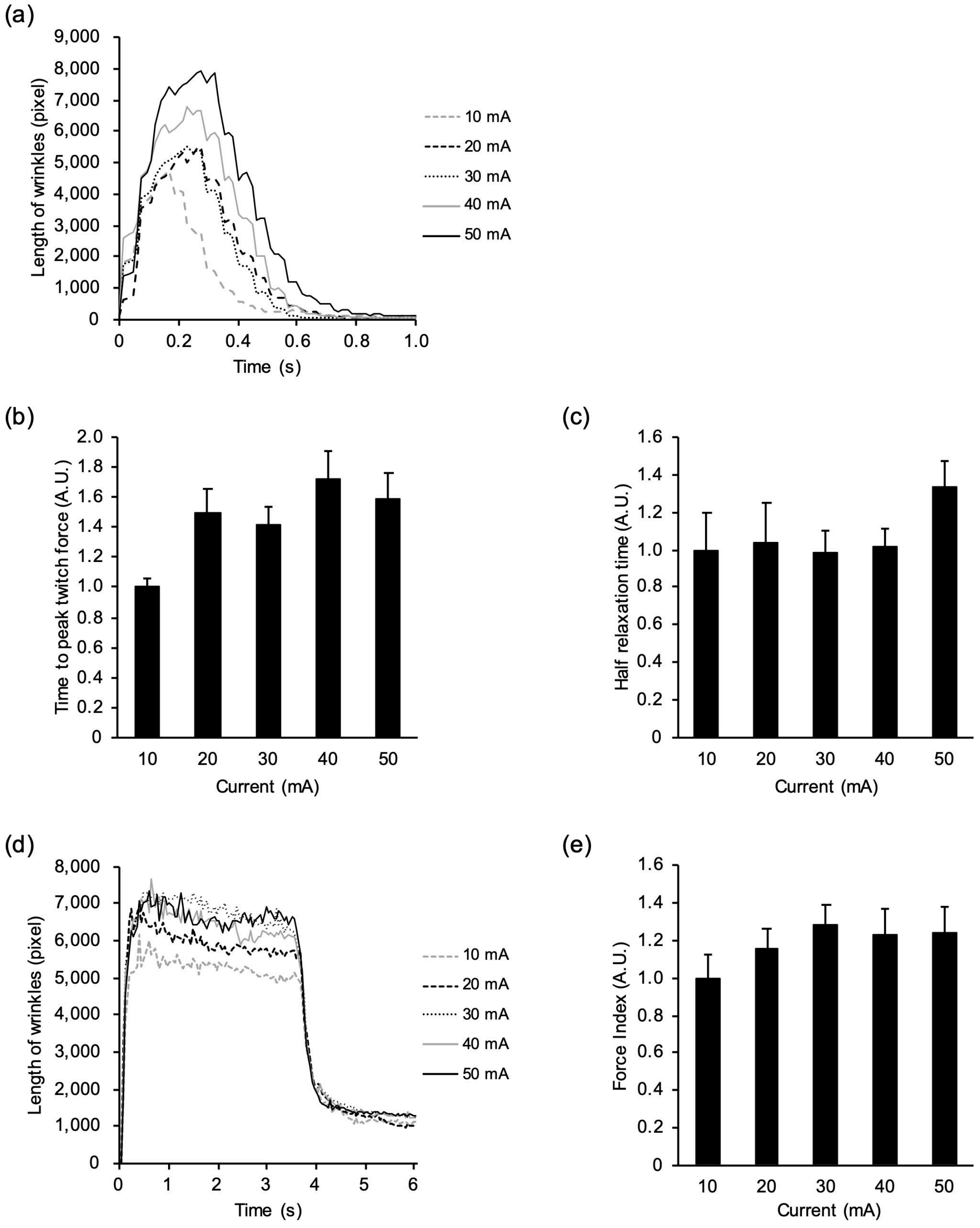

Supplementary Figure 1. Properties of twitch contraction and tetanic contractile force in myotubes with different electrical pulse condition.

(a) The length of wrinkles from the myotube twitch contraction. Myotubes were stimulated with

electrical pulses of 10, 20, 30, 40, and 50 mA at 1 Hz (20 ms duration, 980 ms intervals). Plotting the total length of wrinkles on the substrate generated by the contraction in response to pulses with different currents of electrical pulse. (b) Evaluation of the time to peak twitch force (TPT) and (c) the half relaxation time (HRT) with different currents of electrical pulse. N = 7 (N represents an independent experiment; each "N" shows the average value from 5 areas on the same substrate). (d) The length of wrinkles from the myotube tetanic contraction. Myotubes were stimulated with electric pulses of 10, 20, 30, 40, and 50 mA at 100 Hz consisting of 2 ms duration for 3 s per cycle. The total length of wrinkles on the substrate generated by the contraction in response to pulses with different currents of electrical pulse were plotted. (e) Evaluation of the force index as an index of tetanic contractile force with different currents of electrical pulse. N = 10 (N represents an independent experiment; each "N" shows the average value from 5 areas on the same substrate). Data are shown as mean ± S.E.M. Significance was identified using one-way ANOVA, at $p < 0.05$, followed by a Bonferroni post hoc test.

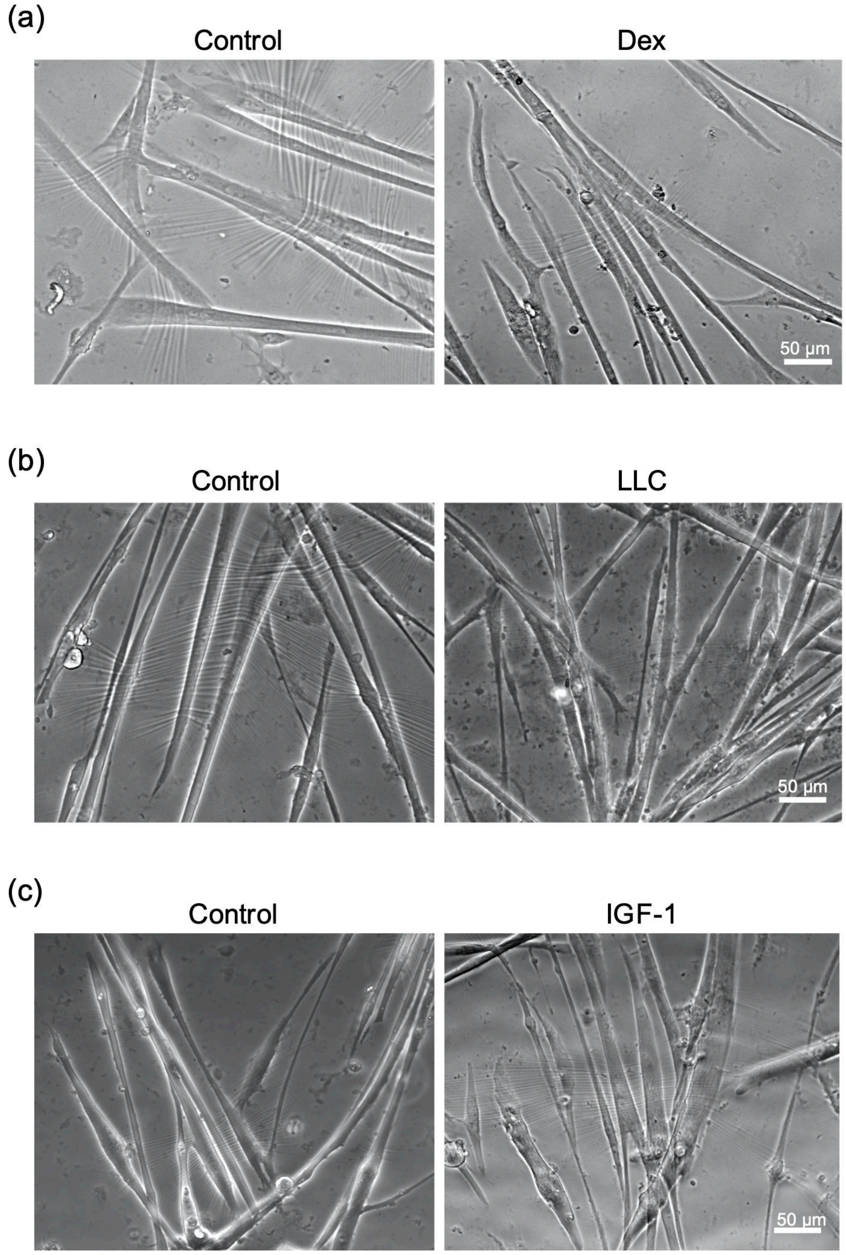

Supplementary Figure 2. Wrinkles formed on the substrate by atrophic/hypertrophic myotubes.

The image shows wrinkles formed on the substrate for the control- and (a) dexamethasone (Dex)- treated myotubes, (b) the conditioned medium of Lewis lung carcinoma (LLC)- treated myotubes, and (c) insulin-like growth factor I (IGF-1)- treated myotubes. Scale bar is 50 µm.

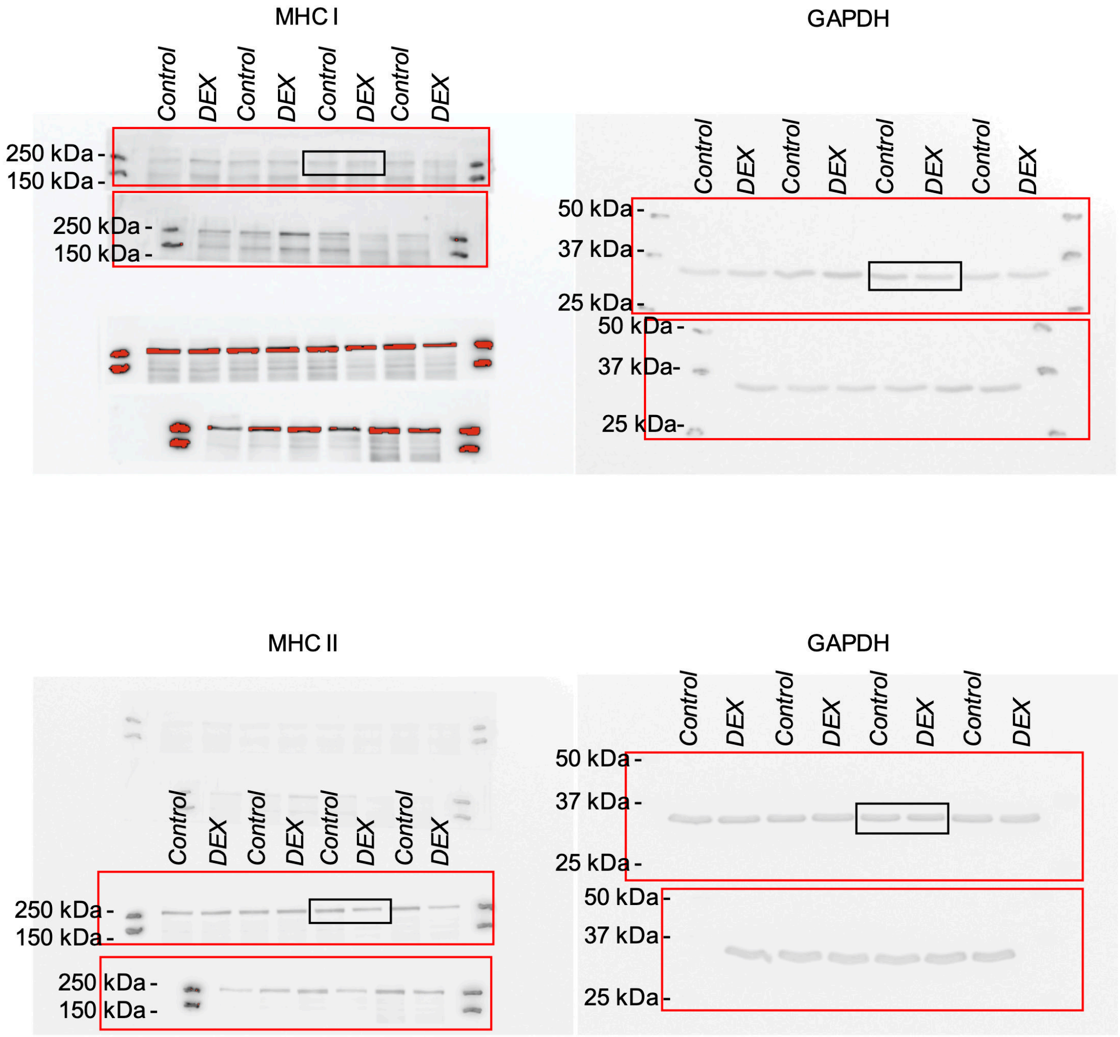

Supplementary Figure 3. Original images of the western blotting in Fig. 5c. The cropped images in Fig. 5c were outlined in black. The over-exposed bands (red) were not used in this analysis. Since the edge in some of the membranes in the images is unclear under this contrast, the edge was outlined in red.

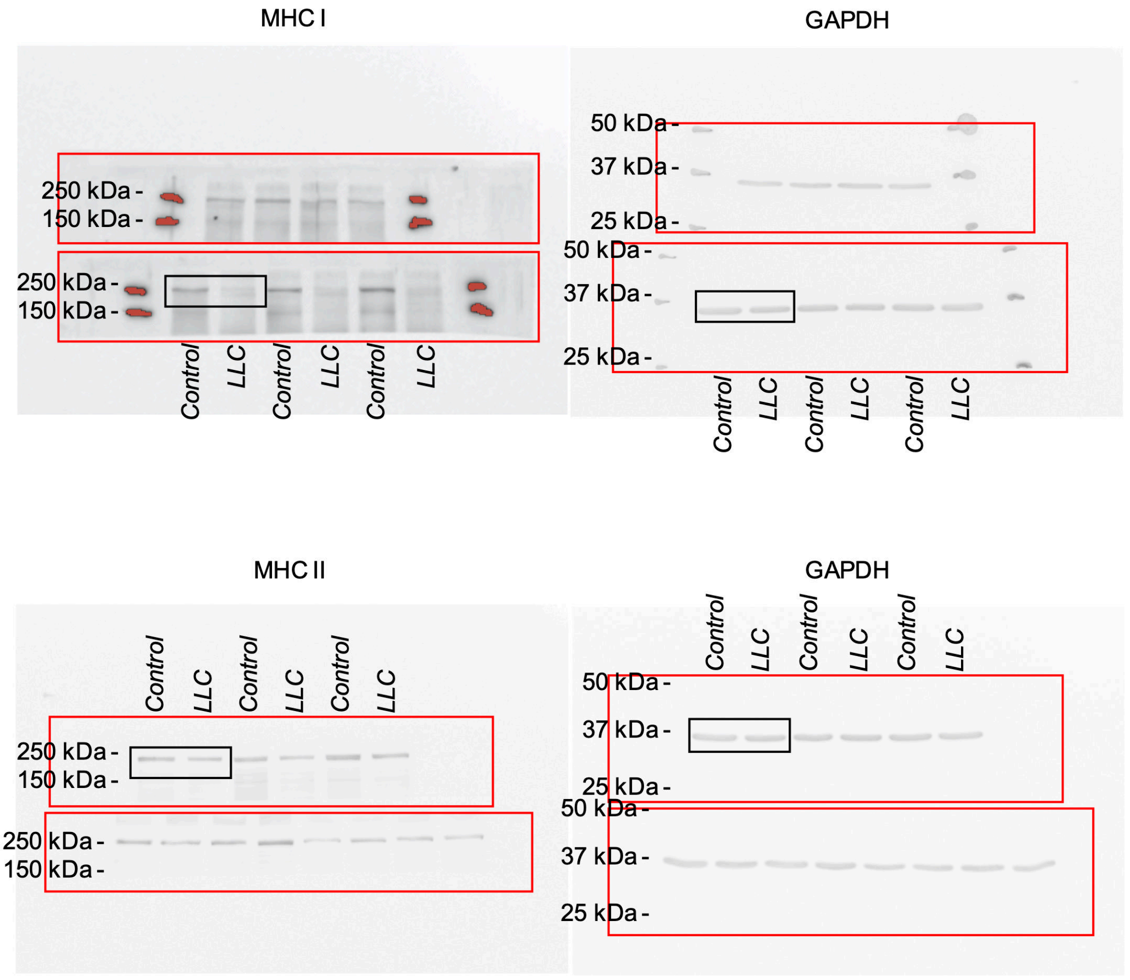

Supplementary Figure 4. Original images of the western blotting in Fig. 6c.

The cropped images in Fig. 6c were outlined in black. Since the edge in some of the membranes in the images is unclear under this contrast, the edge was outlined in red.

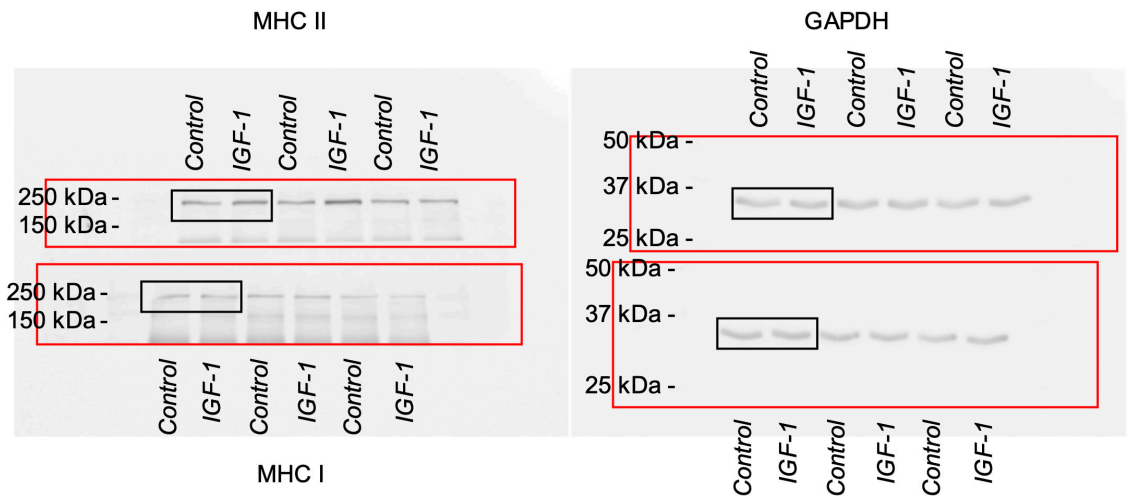

Supplementary Figure 5. Original images of the western blotting in Fig. 7c.

The cropped images in Fig. 7c were outlined in black. Since the edge in some of the membranes in the images is unclear under this contrast, the edge was outlined in red.

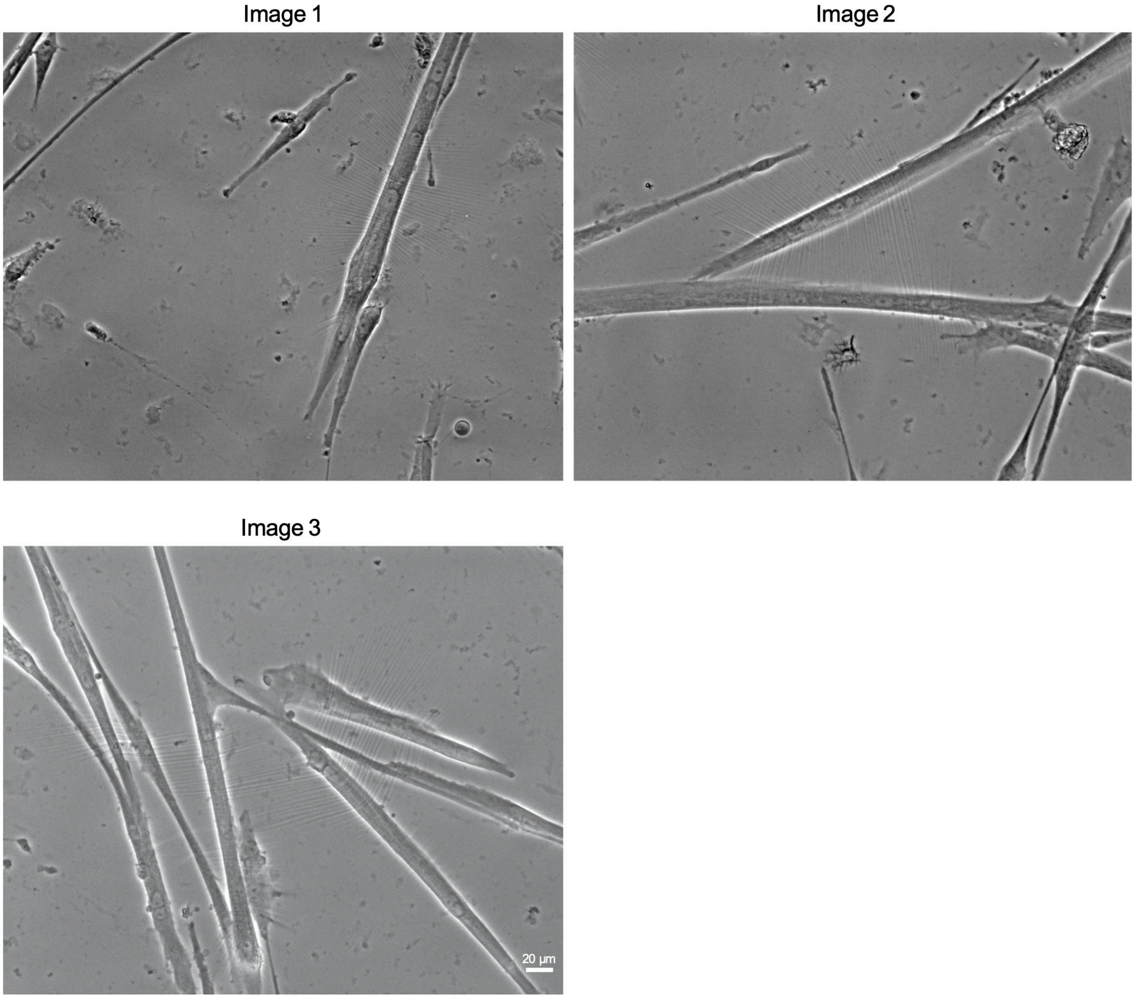

Supplementary Figure 6. Myotube alignment and the number of myotubes are different among the 3 images. Scale bar is 20 µm.

Supplementary Movie

Supplementary Movie 1. Observation of muscle contractile force as wrinkles.

Supplementary Movie 2. Applying force to myotubes generates wrinkles on the substrate.

Supplementary Movie 3. Extracted wrinkles superimposed on the myotubes during contraction. Good correspondence is seen between the automatically extracted lines and the wrinkles in the image.

Supplementary Movie 4. Myotube tetanic contraction. Myotubes were stimulated with electric pulses of 20 mA at 100 Hz consisting of 2 ms duration for 3 s per cycle. The myotubes showed sustained tetanic contraction, and the wrinkles were seen to form and be sustained over time.
