# Supplementary figures and images for: Establishment of a system evaluating the contractile force of electrically stimulated myotubes from wrinkles formed on elastic substrate
Source: Sci Rep. 2022 Aug 15;12:13818. doi: 10.1038/s41598-022-17548-7 (PMC9378739; doi:10.1038/s41598-022-17548-7)

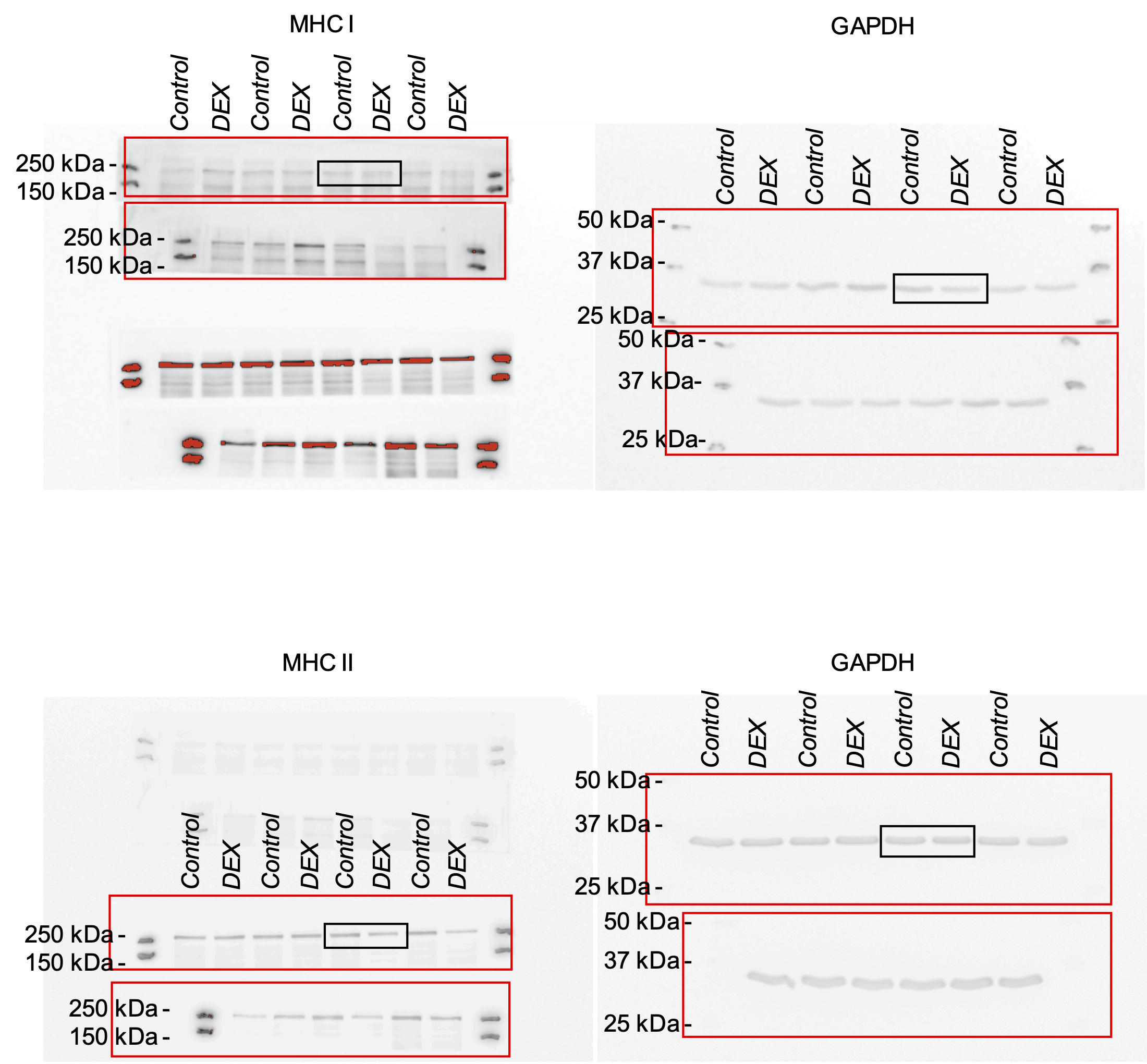

Supplement: Supplementary file 2 — Supplementary Information 2. [file 41598_2022_17548_MOESM2_ESM.png]

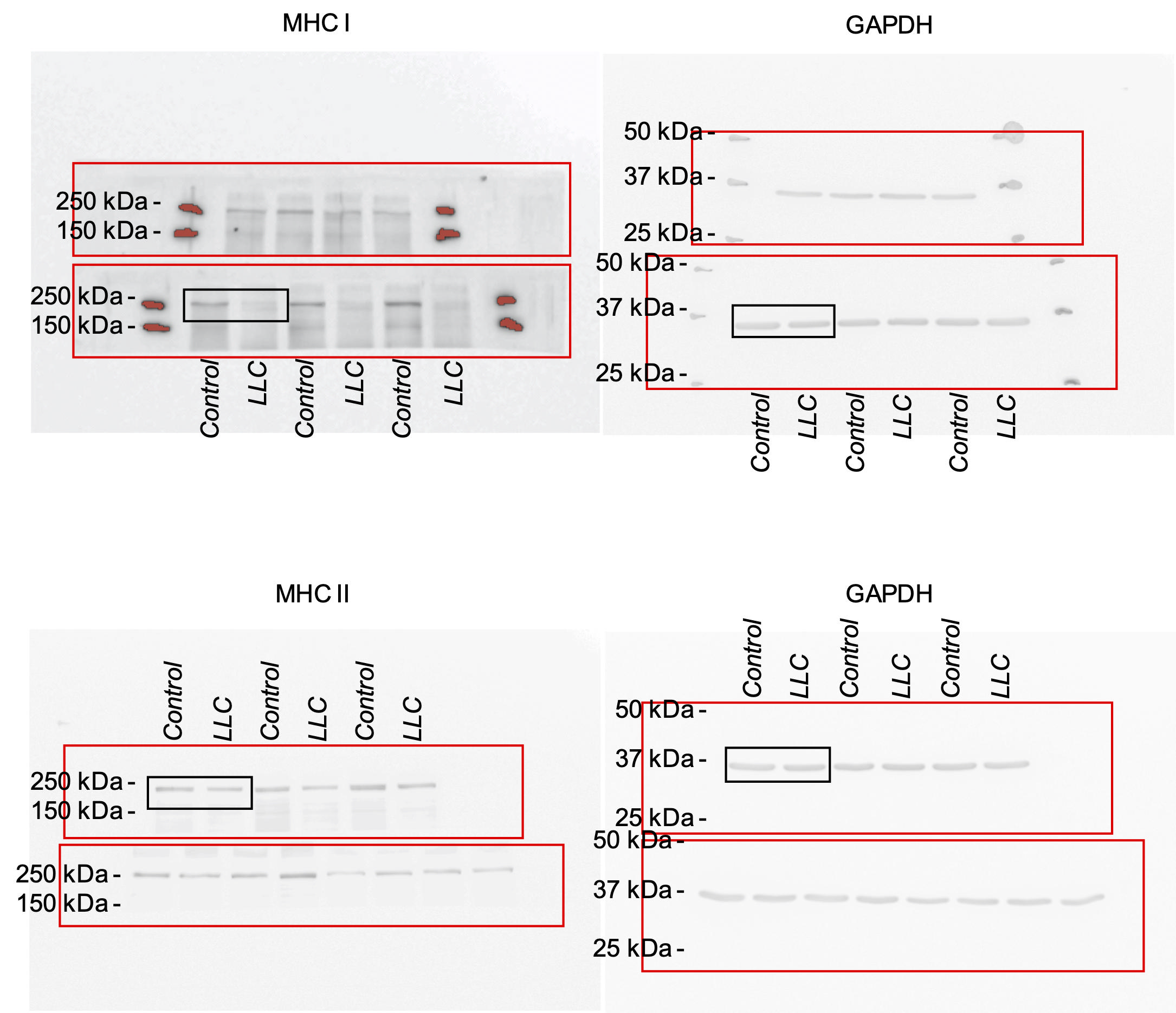

Supplement: Supplementary file 3 — Supplementary Information 3. [file 41598_2022_17548_MOESM3_ESM.png]

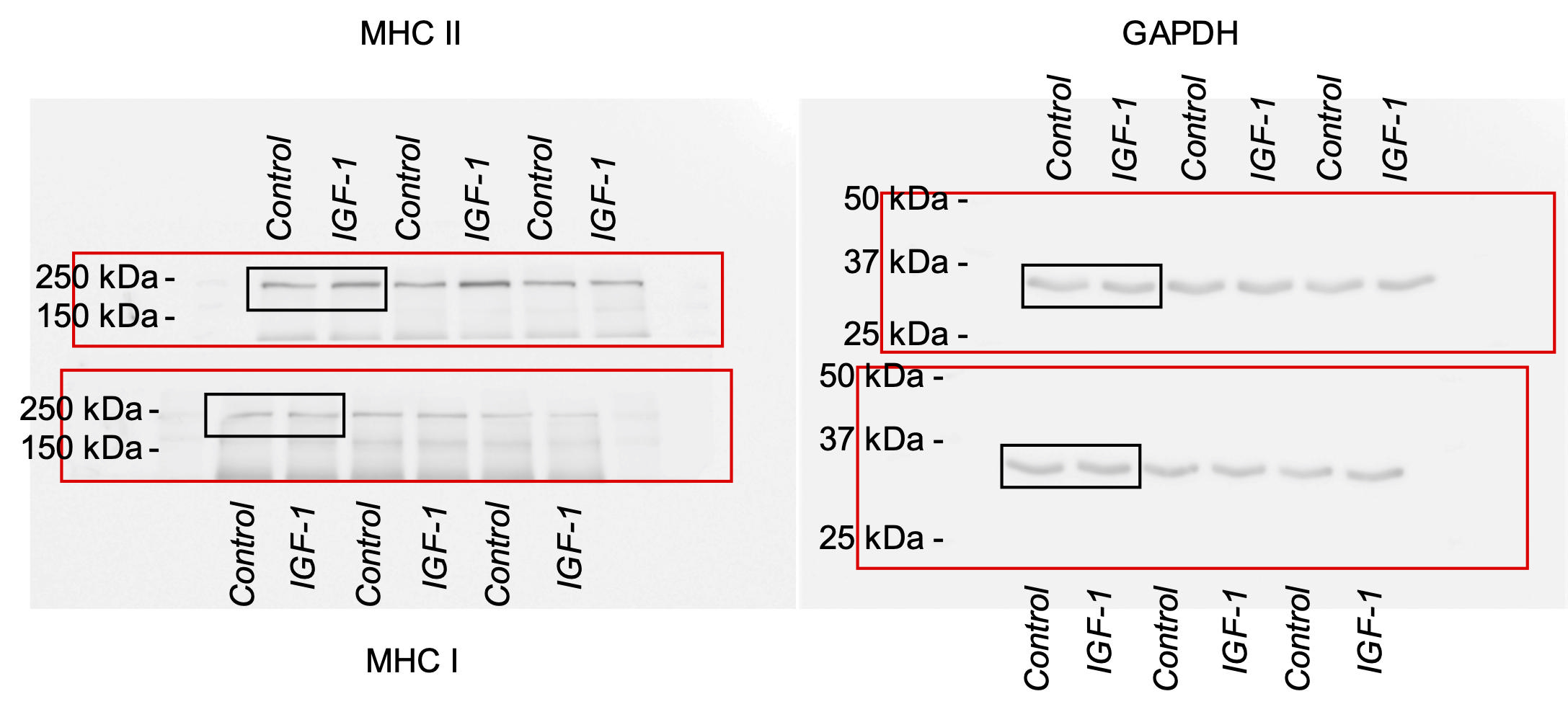

Supplement: Supplementary file 4 — Supplementary Information 4. [file 41598_2022_17548_MOESM4_ESM.png]
